# Mr. Annesley on the Diseases of India

**Published:** 1828-04-01

**Authors:** 


### Medico-Chirurgical Review,

### No. XVI.

[FASCICULUS VI.]

MARCH 22, 1828.

### Art. XV.

*Researches into the Causes, Nature, and Treatment of the most prevalent Diseases of India, and of Warm Climates generally. Illustrated with Cases, post-mortem Examinations, and numerous coloured Engravings of Morbid Structure.* By James Annesley, Esq. of the Madras Medical Establishment, &c. &c. Imperial Quarto, pp. 700, with 21 coloured Plates. Vol. the First. Longman and Co. 1828.

[Art. I.]

THIS magnificent work will transmit Mr. Annesley's name to posterity, in conjunction with the medical history of our extensive empire in the East. We know not which to admire most—the indefatigable labour, and the unconquerable zeal of the author in the collection of his facts, or the beauty and fidelity of the plates, which portray the ravages of disease as it appears in the Torrid Zone, with the most scrupulous accuracy.* We hope and trust that the East India Company will do an act of justice in rewarding Mr. Annesley for the toil of mind and body which he must have undergone in the construction of this immense undertaking—leaving the tremendous expenses out of the question. If they can so cheerfully vote away their thousands in the annual pension of those who make war—they surely might well expend a few hundreds in the encouragement of those whose labours will mitigate the miseries of warfare and the deleterious influence of climate, long after their bones are mouldered into dust.

We do not deem it necessary now to apologise for dwelling on those diseases which scourge our countrymen beneath a foreign and burning sky. Some of the greatest improvements in medicine have resulted from researches made in hot climates—and there is not a single fact observed, or a single disease inves-

* It will doubtless be said that some of the plates are too highly coloured. This may be true—and it is an objection to most coloured plates. But it should be remembered, that diseases run a rapid course in high temperatures, and that dissections are necessarily made there, in a few hours after death.

tigated on the banks of the Ganges or the Mississippi, that does not bring its quota of utility to the practice of medicine in our own country.

After stating the excellent means which he possessed, for a great many years, in India, of acquiring and registering the most authentic information, Mr. Annesley remarks as follows:—

"In India, the medical practitioner has every possible opportunity of investigating disease by *post mortem* examinations, and of connecting the symptoms and treatment with those morbid changes which take place in its course. To this subject the Author has always paid especial attention: but the great difficulty of describing morbid structures, and the impossibility of preserving the natural appearances in the way morbid preparations are usually made, led him to cause Drawings to be executed of the more interesting and remarkable changes produced upon the internal organs by the diseases he was called upon to treat. Circumstances placed in his power the means of accomplishing this object, and he fully availed himself of them. *Post mortem* examinations necessarily take place in warm climates soon after death, and before the capillary circulation in the internal organs has undergone that change which is experienced after a few hours, or before the blood has returned from the minute arteries into the venous trunks. Thus, the warmth of the climate has indirectly enabled him, it may be presumed, to give a more correct delineation of the appearance of diseased structure than could otherwise have been obtained. The knowledge unfolded by this circumstance induced him to follow up the indications to which it pointed; and as an early examination of the subject of disease after death appeared necessary to accurate ideas as to the more minute changes and finer shades of disorder, impressed upon the different internal viscera during life, it was never neglected when it could be practised with propriety."

This is a very important consideration, and tends to enhance the value of the work under review. We must pass over the preliminary discourse of our author, in which he dwells, with allowable earnestness, on the advantages which a long residence in India has conferred on himself—and points out, in no very measured language, the sources of error which may have operated, where the residence has been short, the scene of observation limited, or the constitutions of the patients of a peculiar description. All these things we are ready to grant to our experienced author; but we would just hint, from some 30 years close observation of men in all climates, that it is a comparatively rare occurrence to find any discovery or improvement in medicine result from *mere length of experience.* We refer Mr. A. to the history of our art generally—and to the medical history of tropical climates particularly, for the proofs of this position. Mr. A. regrets that few or none of the old and experienced practitioners of India have left us any records of their practice. If we look to the West, we shall see the same thing. Have any of the old residents of the Antilles left us any works to compare with those of Jackson and others, who were only a very few years in that unhealthy climate? In short, unless observations are made in the vigour of life, by medical men in hot climates, they will never be made at all! After a certain number of years, in hot as well as in cold climates, the current of zeal, in the minds of medical practitioners, is too often dried up or frozen up—and it is replaced either by a sharp look out for the "main chance"—or a settled resolution to take things easy—to enjoy the short span of existence with as little encumbrance as possible—and to leave to others the trouble of observing for themselves, as they themselves were obliged to do! Mr. Annesley, it is true, forms an exception to the general rule. He is a "rara avis in terris"—but he may take our word for it, his call on the old practitioners will meet with few responses. Let us, therefore, be thankful for the contributions of our younger brethren, both here and elsewhere—and let us be doubly thankful to such men as Mr. Annesley, who have devoted a long series of years to practical and pathological researches beneath the enervating influence of a vertical sun, and surrounded by so many temptations to indolence and luxury!

But, leaving these considerations on one side, we come to the work itself. We must pass over the whole of the first chapter of the work, occupying 45 folio pages, which treats of the physiology of digestion, and the functions of the liver, spleen, &c. Considering what a size and price Mr. Annesley's book necessarily amounted to, we cannot but think a chapter on the mere elementary matter of digestion, to be found in the school-books already in the possession of even pupils, was somewhat impolitic. We next come to the second chapter, exhibiting "a general view of the causes chiefly productive of diseases in warm climates, particularly in India."

### Malaria.

This subject has been treated in so masterly a manner by Dr. Macculloch, that little or nothing new or interesting can be expected from Mr. Annesley. The copious analysis which we have given of Dr. M's work will be a sufficient excuse for passing over the section in question from the pen of Mr. A. We shall notice one particular only. The writers on marsh miasmata have generally insisted, especially since Dr. Bancroft's Essay appeared, that animal matters have nothing to do with the poison of terrestrial exhalations. We have always been of a different opinion, and the following passage shews that we have Mr. Annesley on our side.

"A most important circumstance, which goes far to account for the much greater unhealthiness of moist and marshy situations in warm countries, is the quantity of animal matter, in a state of decomposition, which they present. The same circumstances which render vegetation quick and luxuriant, tend also to generate immense swarms of reptiles and insects; the exuviæ and dead bodies of which, mingling with vegetable matter in a state of decay, and combining with moisture, give rise to miasms of a much more noxious description than those resulting from vegetable decomposition and moisture alone. In the course of our experience in warm climates, we always have considered the number of insects and reptiles with which a place abounds, as more indicative of its unhealthiness than any other circumstance; for in it there is a most powerful cause of disease in its worst forms superadded to those already in existence; and, as the one cause is extensive and powerful, so, generally, is the other. The great unhealthiness of low, moist, and marshy places in temperate climates, during warm seasons, particularly in the months of July, August, September, and October, is as much owing to the immense swarms of insects which then abound, and which die during these months. Italy furnishes numerous proofs of this; and every warm country in the globe will verify the axiom, that a place is unhealthy in proportion as it furnishes, with the various causes of disease depending upon locality and temperature, animal remains and animal substances in a state of decomposition, mingled with the products resulting from the decay of vegetable matter."

In a subsection, Mr. Annesley introduces some remarks on the nature, properties and effects of miasmata—and on the manner in which they invade the system. In respect to the *first* point, it is hardly necessary to say that we know nothing. We are as ignorant of the nature or essence of malaria as we are of the inhabitants of the Georgium Sidus. Of the properties and laws of vegeto-animal effluvia, we have taken ample notice in our review of Dr. Macculloch's Essay—and Mr. A. appears to have drawn pretty feely on that and all preceding works on malaria. We shall proceed at once to "the effects of malaria on the human constitution." Intermittent and remittent fevers, of course, are the acknowledged products of the invisible poison—"even yellow fever, in its worst forms, seems to be the consequence of these causes operating in a state of greater activity or concentration upon highly disposed subjects." Mr. A. believes the plague of Egypt to be the product of malaria. Next in importance, to fever, is dysentery, which, in its epidemic forms, Mr. A. ascribes (and probably with justice) to malaria. He does not deny, however, that sporadic cases of this disease are the products of vicissitudes of temperature, errors in diet, intoxication, and other causes. He thinks there can be no doubt that the scorbutic dysentery, so well described by the late Mr. Bampfield, is produced by terrestrial exhalations acting on constitutions badly nourished by improper food. He says the scorbutic dysentery which prevailed at Rangoon, and the endemic at the Milbank Penitentiary, are recent examples of this. Even hepatitis he is disposed to attribute to malaria conjoined with tropical heat. "There is seldom seen, within the tropics, a case of disease in which, upon dissection, the liver and spleen are both sound." In fevers from marsh effluvia, indeed, whether within or without the tropics, there seems to be a strong tendency to derangements of the liver and spleen. The exposure, therefore, to malaria, even when no fever or dysentery is produced, seems to affect the hepatic system, as we see in all marshy countries. The malaria of India has an infinitely greater effect on the European than on the native population. If the children of Europeans are not sent home young, their constitutions are liable to be ruined, and the range of their existence abridged.

"In addition to the diseases we have enumerated as being produced among Europeans by malaria, and in addition to its blighting effects in warm climates upon a native white population, even when it fails of inducing active and specific disorder, we should particularize its influence in occasioning ulcers of the lower extremities, and foul sores, and even sphacelation and gangrene. Every military surgeon has numerous opportunities of observing, in the East, the relation which subsists between unwholesome situations and these disorders, both among Europeans and natives. Indeed, it seems to be a general and necessary effect of malaria to diminish the powers of life throughout the whole body; and the phenomena accompanying and indicating this effect are various, according to numerous concurrent circumstances, to predisposing causes, and to concomitant influences. Of these we shall have occasion to speak when the diseases proceeding chiefly from this grand agent come specifically before us."

In respect to the modus operandi of malaria, or even the channel through which the poison is conveyed, Mr. A. is unable to furnish us with any positive information.

"But observation has supplied data, which, when calmly considered, seem to shew that terrestrial emanations, and all those causes of disease which float in the atmosphere, make an impression on those surfaces with which the air comes in contact: and this impression, when sufficiently strong, or frequently made, is productive of disease, either of the system generally, as in fever, or of some important viscus, as the liver or spleen. It is, therefore, chiefly to the internal surfaces of the lungs and air passages that we are to look as the channels through which malaria makes its hurtful impression upon the animal frame. But whether it acts by deranging the healthy condition of the nervous system of the organ, which derangement produces farther disorder until specific disease is fully formed; or whether the exhalations floating in the air are actually absorbed from the surfaces of the air passages and cells into the blood, vitiating this fluid, and, by its presence there, deranging the whole system, or some important viscus, it is impossible to decide. Both sides of the question have found supporters who have adduced arguments in behalf of their opinion, in the absence of positive proofs."

Mr. Annesley seems to conclude that both may be combined. The miasm may offend the nervous system, and, entering the circulation, may vitiate the fluids at the same time.

After taking a survey of those circumstances which favour the operation of malaria, as irregularities of all kind—and more especially the depressing passions, indolence, &c. our author devotes a subsection to the consideration of the means of preventing malaria, and counteracting its effects on the human body. These subjects have been amply discussed—or will be so, in our reviews of Dr. Macculloch's volumes. We must pass over another great portion of the work before us, consisting of medical topography—embracing both hemispheres, as well as the Mediterranean. From a section on the diet of Europeans in India, we shall select the following passage, exhibiting the "diary of a day," if we can use such an expression. It is a matter of curiosity, in more than one respect.

"The military officer goes to parade at six o'clock A.M., and breakfasts between eight and nine upon tea, coffee, or cocoa, with fish, meat, eggs, rice, and whatever may be most agreeable to him. From breakfast till one o'clock he generally applies to study or amusement, or to paying visits. The heat of the weather, and perhaps a hearty breakfast, and the nature of the articles taken at it, produce thirst, which renders the necessity of gratifying it urgent, and occasional draughts of wine and water, beer and water, or brandy and water, are therefore necessarily taken; and although this is by no means a habit, nor is indulged in beyond what seems a matter of necessity, yet it must, in a certain degree, be injurious. At one o'clock he eats a hearty tiffen, consisting of roast and boiled meat, fish, mullagatawny or other soups, various wines, bottled beer, &c. He afterwards occasionally rides out in the sun, and either lounges on a sofa, or amuses himself with cricket or fives till evening parade. Dinner is next disposed of, at seven o'clock, or half-past seven, or eight. This meal is, properly speaking, the supper, that which is taken at one o'clock being the dinner. The seven o'clock meal is generally profuse, consisting of soups, fish, rich and hot curries, roast and boiled meats, and other richly made dishes, with various wines, and bottled beer. To all this succeeds coffee or tea; and upon the repleted stomach and excited system he retires to bed at eleven or twelve, when the feverish collapse induces the sound sleep indicating plethora, or the restless slumbers attendant upon prolonged excitement."

The above is a faithful diary of a tropical sojourner in the military service, and we have reason to believe that it equally applies to the civil servant of the Company. This being the case, we cannot wonder that our countrymen return from the East more frequently with enlarged livers than lacks of rupees. It is seen that animal food is partaken of largely, at least twice, but generally thrice in the day, together with an abundant supply of various other stimuli and provocatives—all this, too, in a climate where adequate corporeal exercise cannot be taken, without immediate risk of life. That such a system of repletion must keep up a constant over-excitement in the digestive organs, including the liver and spleen, cannot be doubted. It would do so beneath the gloomy skies of England, with all the exercises of the field, and the bracing air of a British winter! Mr. Annesley protests against this system of full living, and eloquently supports the arguments of those who have gone before him in this line of investigation. We cannot so fully concur with our talented and experienced author in his ideas respecting exercise in tropical climates. Mr. A. strenuously recommends corporeal exercise "so as to promote a full and copious perspiration and regular circulation in the cutaneous surface." We are advocates for exercise, in all proper times and places; but we have been led, from personal feeling as well as observation, to regard corporeal exertion as much less adapted to tropical than hyperborean regions. The vessels of the surface are too much excited by the heat of an Indian atmosphere, even when a person is at rest, and therefore, we believe that quietude in the middle of the day is best, while we recommend exercise before sunrise and in the evenings, in moderation. This, indeed, after all, is the rule to which Mr. A. comes in the sequel.

In a chapter on the premonitory symptoms of diseases, we find many judicious observations. Every one knows that there is an interval between the application of a morbid cause—say the cause of fever, and the development of its effects. This may be termed the period of incubation. The phenomena which take place in this interval generally pass unnoticed, or unattended to by the patient; but a careful observer will see that the seeds of disease are sown, and that a storm is impending.

"In fuller illustration of this subject, we shall instance a very frequent case, and one that will be recognised, not only by those in India, but by those who have ever been there;—we allude to snipe shooting and hunting parties. These are generally arranged late in the evening, after dinner, and are entered upon early in the morning.

It is impossible, therefore, that the individuals engaged in them can have the repose necessary to recruit the system from the exertions of the preceding day. After riding eight or ten miles, they commence snipe shooting in the marshes and rice-fields, where they are up to their knees in water; and thus, in a state of fatigue, they are at once brought within the influence of those marshy exhalations which are the most frequent exciting cause of fever in warm climates. The exposure to this cause taking place during a period of predisposition to its invasion, and at a time of the day when the cause itself is in considerable concentration, that impression is made upon the system which is productive of fever, and its future subject returns from his excursion with the seeds of it sown in his frame. For a day or two he complains of little or nothing excepting a weight in his back, loins, and limbs, some loss of appetite, and a disinclination to exercise or employment of any kind. To these he attaches no importance, imputes them to fatigue from his excursion, and he does not resort to any means for removing them. They, however, continue, and even increase; and in a short time a slight headach, with confusion of ideas, comes on, especially towards evening, and is attended with disturbed repose and unpleasant dreams. His appetite now becomes further diminished, his countenance is pale, sallow, and a somewhat darker tinge is remarked beneath his eyes, which are at the same time muddy, and deficient of their usual expression and liveliness. These symptoms continue for several days: they are insufficient to confine him, or even to excite ideas of his being actually ill; but he feels out of health, and every kind of occupation is a burden to him. At last, after a period widely varying in its duration, generally enduring from two or three days to a fortnight,—during which time these symptoms continue gradually to increase,—nausea often supervenes, the bowels become irregular, the tongue white and loaded, the countenance sunk and muddy, the surface cold, dry, and harsh; and at last, irregular chills, formication, and even complete rigors, supervene, with sinking and a sense of anxiety at the pit of the stomach and præcordia, and increase of the pain in the head, loins, and limbs. This is that precise stage of the disease at which the patient generally becomes alarmed, and when he is first unable to keep about."

When we carefully consider the foregoing phenomena, which are correctly recorded, we will be constrained to admit, that they afford full as much support to the humoral, as to the nervous pathology of diseases. The sallow countenance, the dark tinge round the eyes, the white and loaded tongue, together with the deranged state of the secretions and excretions, which the author has not sufficiently noticed—all these are as indicative of vitiated fluids, as of disordered nerves in the body. But, on this subject, we have already dilated in another part of our Journal. Mr. A. goes on to state some causes of disease in warm climates, which are of considerable importance. The mode of living before-described, combined with want of exercise, tends to plethora, and this is aggravated by a constipated state of the bowels, and a vitiated condition of the secretions. These circumstances, our author observes, "tend very rapidly to vitiate the constitution of the blood itself." After making many judicious observations on the state of various functions, as indicative of incipient disease, Mr. A. comes to the second book of his work, in which he takes up the subject of disorders of the stomach, as they appear in tropical climates.

### STOMACH DISORDERS.

Our author remarks, that this class of complaints is comparatively rare in hot climates, at least in "a pure and uncomplicated form." They are seldom much noticed, till they become connected with, or give rise to, more serious disease, as of the liver or intestines. Mr. Annesley does not profess to go very deeply into the investigation of stomach disorders, and we are forced to confess, that the short dissertation contained in the work evinces neither novelty nor originality. Our author is disinclined to view, as some have done, the stomach affection as the *cause* of the biliary derangement. The causes of both these complaints, he properly observes, are simultaneously acting on the two organs, and although the first symptoms are generally noticed in the process of digestion, yet it does not follow that disordered biliary secretion is behind hand in its part of the morbid process that is going forward. We are unable to glean any thing from this chapter, and shall, therefore, pass on to that "on inflammation and organic lesion of the stomach." Mr. A. remarks, that simple and uncomplicated inflammation of this organ is an extremely rare disease in tropical and in cold climates. But the mucous coat is very frequently inflamed secondarily, between the tropics, in consequence of the extension of disease from the liver or other contiguous viscera. Mr. Annesley appears to place faith in the doctrine of Dr. Philip, that the advanced stage of indigestion is that of inflammation. The mode in which he conveys his belief is rather equivocal. "It (inflammation) *supervenes*, we are most thoroughly convinced, to a greater or less extent, in the advanced stages of dyspepsia." This may be the case, but this does not prove that inflammation is the *cause* of the disease. The following short case, which we shall extract verbatim, from page 253 of the work before us, will shew how far the treatment of stomach affections needs reformation in the East.

"CASE.—*Inflammation of the Mucous Coat of the Stomach supervening to Dyspepsia, and terminating in Ulceration.—Dissection.*

"WILLIAM SPARKS, admitted 19th of June, 1815.—Returned from field service much emaciated, and extremely languid. Has been complaining for some time of dyspeptic symptoms, with occasional attacks of fever. Countenance sallow; tongue foul; some purging, without pain; no fulness in the hypochondria; pulse 78; skin warm and moist. Has used wine with tonics.—℞ Mist. amaræ, (infusi gentian. comp.) ℥j.; tinct. cinchonæ, ʒij.

"22d.—Bad taste of the mouth, with nausea and general sickness; no desire for food; bowels rather open.—Capiat Pulv. ipecacuan. ℈j. pro emetico. Take at 11 o'clock, A.M., Mist. amaræ, ℥jss.; tinct. ferri mur. ♏xij.

"*Evening.*—Threw up some dark, grumous fluid after the emetic; several stools.—℞ Aq. menth. pip. ℥jss.; tinct calumbæ, ʒjss.

"23d.—Feels pretty easy this morning; pulse is slow and languid.—℞ Infus. gentianæ comp. ℥jss.; tinct. ferri, ♏x. twice a day. Continue the wine.

"24th.—Repeat the draughts twice a day, and wine.

"26th.—Takes his draughts and wine; appears to be declining; debility increases; bowels loose. Continue draughts and wine.

"*Evening.*—Seems much worse; some hiccup, and the pulse at the wrist nearly gone; debility extreme. Continue the wine.—℞ Mist. camphorat. ℥jss.; spirit. æther. nitros. ʒij.; aquæ, ʒvj. M. statim. Blisters to the insides of the legs.

"27th.—Died this morning.

"*Dissection.*—The coats of the stomach were much thinner than natural; the villous coat was found covered with small and numerous superficial ulcerations, which were still more numerous near the cardiac orifice; they discharged a thin brownish fluid, which was very fœtid; the liver and spleen were sound. The small and large intestines appeared healthy."

Mr. Annesley condemns the practice in this case—and we have no hesitation in affirming, that "the inflammation supervening to dyspepsia," in this case, was induced by the treatment, and had no necessary dependence on the natural progress of the disease itself. But we cannot dwell on the stomach any longer, leaving the disorders of this organ to Abernethy and his followers; while the pathology will be best studied among Continental writers, who pay more attention to dissection than those of our own country.

### Diseases of the Liver and Biliary Apparatus.

It has often been remarked, that hepatitis is much more frequent in the Eastern, than in the Western Tropics. The returns of regimental sick shew that it is at least treble in the former to what it is in the latter. In India, the average annual per centage of liver-complaints, in the different divisions of the army, was estimated at 13 per cent. in the effective strength!

The increased secretion of bile observable in Europeans on their removal to a tropical climate, has been ascribed, by Dr. Johnson, to a sympathy between the skin and the internal organs. The sympathy is admitted by Mr. Annesley, but the doctrine is superseded by one first discovered by Crawford, Lavoisier, &c. and confirmed (as is said) by Dr. Copland, on the coast of Africa—namely, "that the quantity of carbonic acid gas, formed by respiration in a given time, is much diminished in high temperature, and under circumstances which lower the powers of life." This being established, they say, (for it is presumable that Dr. Copland speaks, together with the author) "it becomes a basis on which much *important speculation* respecting the origin of several intertropical diseases may be founded." Thus the diminished formation of carbonic acid gas in the lungs, during a high temperature, must increase the secretion of bile, and so on. We do not deem it necessary to discuss this chemical physiology. There is one little fact past over by the ingenious authors, which, in our humble opinion, proves fatal to this gazeous hypothesis. It is this. After a certain residence in hot climates, the torpid state of the liver is just as evident as the increased function of that organ was in the early residence. Yet the temperature of the tropics does not, we believe, alter, so as to accommodate the theory of one or other party. This fact does not militate against, but rather supports, the doctrine of sympathy between the skin and liver. In the long residenters, when the biliary organ falls below par in function, the skin is in an analogous state—dry and constricted. Our authors have wasted a great many royal quarto pages in the pursuit of these theoretical phantoms, instead of filling them with practical facts, that might turn to better use.

A number of common cases of increased secretion of bile, among newly arrived Europeans, are detailed with much minuteness, and then the treatment of "increased secretion of bile" is entered upon. With every disposition to be pleased with the work before us, we cannot help being grieved to see the systematic author so much predominate over the practical writer. By this procedure, the work has been rather injured, than benefited, for general circulation and use. We could substantiate this position by many extracts. The following quotation, in which the *dilution* of facts by words is really less than in almost any part which we could possibly pitch upon, will suffice.

"During our practice in India, we have had numerous opportunities of observing, in the *post mortem* inspection of those who had died of diseases either immediately seated in the liver, or affecting other organs, the gall-bladder distended with a thick, viscid, and acrid bile, and the ducts running from the secreting granulæ of the liver through its substance to their principal trunk completely gorged with bile of nearly similar characters. In different cases, indeed, this secretion presented different appearances, as regards colour and consistence; but the engorgement of the ducts and gall-bladder was generally remarkable, without any apparent organic change sufficient to account for the circumstance. In the majority of instances, the outlet of the ducts in the duodenum was quite free, and their channels unobstructed, unless the viscidity of the secretion may be viewed as an impediment,*—an inference that seems by no means irrational. Where any obstacle existed, such as narrowing of the ducts, the impaction of calculi in them, or the existence of spasm,—the cause was then evident; but in the absence of all these, the only conclusion we could form as to the cause of this very frequent appearance, was, that the secreting functions of the liver may be so modified in a warm climate, that, in addition to an increase of the biliary secretion, this fluid itself may be retained and accumulated in those parts of the apparatus which admit of the retention. Attentive observation of the phenomena, marking the origin and progress of the diseases of the liver and bowels, and of the various types of fever, has further confirmed our opinion as to this particular point, and convinced us that this state of function actually obtains at the commencement and during the progress of these disorders, more frequently than is supposed, and is actually oftener present at these periods of ailment than in the last or fatal stage of disease; and that it is not only met with as a symptom or concurrent phenomenon in these disorders, but as an ailment *sui generis*, the disturbance observed in the system being the result of this cause, or arising from the irruption of the long-retained bile into the alimentary canal.

"During an increased secretion of bile, if any momentary impediment come in the way of the flow of this fluid, either in the course of the common duct, or at its outlet, a copious regurgitation of it into the gall-bladder, and accumulation of it in the biliary ducts, must be the consequence; and if the obstacle placed in the way be either partial or complete, or of short or long duration, the accumulation will be in proportion to its extent and duration, and the copiousness of secretion. If the secretion be going forward abundantly, an obstacle, partial in its operation and of short continuance, will give rise to a great accumulation in the gall-bladder, and in the liver itself. If the secretion be natural, or even less than natural, a more com-

* "We have frequently seen, upon the examination of bodies which had died of different diseases, the gall-bladder loaded with bile of a dark-green colour, and so thick and viscid, that it could scarcely be forced through the ducts by squeezing the gall-bladder, although a blow-pipe or probe would pass readily along them, shewing that the obstruction was then owing to viscidity alone. Doubtless, spasm, or other more permanent obstruction, will frequently arise, as we shall have occasion to shew in the sequel."

plete or long-continued impediment opposing its discharge into the duodenum will have a similar effect. Thus, in recruits and other strangers to the climate, on their arrival in India, when the biliary secretion is much increased, *the temporary obstruction produced by exposure to currents of cool air, to wet, and by eating indigestible and hurtful substances, &c. often occasion the most formidable symptoms of disease, and when the obstruction is overcome, an immense quantity of vitiated bile is passed.* On the other hand, temperate persons, of regular habits and good conduct, are not so liable to these kinds of derangements, and suffer less severely from them when they occur. It is also reasonable to suppose, if the gall-bladder and ducts be over-distended with the accumulation of bile within them, that their vital contractility may be weakened, and that they will be the less able to re-act upon the distending power; and thus the evil will be increased, until that degree of constitutional disturbance be excited by the morbid distension, or until some internal or external cause supervene, which shall enable the organ to throw off the load which oppresses it, and discharge its morbid secretions."

To say nothing of the vague reasonings in the foregoing quotation, what, we ask, becomes of the carbonic theory about the *increased secretion* of bile from atmospheric heat? Here we have currents of *cool air, wet, &c.* not checking the secretion, as it ought to do—no, it has not the least effect of that kind—it only checks, by some theory unexplained, the *exit* of the bile from the liver and gall-bladder. Now, the plain matter of fact appears to be, that the exposure to wet and cold checks both the perspiration and biliary secretion, and when reaction takes place—in other words, when these secretions are restored, there is a redundancy of both fluids, as a necessary consequence. We think it would be difficult to adduce a passage from any work, so pregnant with gratuitous assumptions as the following.

"The obstructions which generally *occasion accumulation of bile* in the apparatus concerned in its secretion and discharge, seem to be whatever suddenly *diminishes the vital influence of the organ* or the system generally; as exposure to terrestrial and morbid exhalations, sudden chills, the depressing passions, the use of cold fluids and ices when the skin is perspiring, &c. Spasm of the common ducts may arise from these and other causes, and produce more completely the same effect. *A weakened state of the digestive organs, particularly of the duodenum and stomach, may also be productive of accumulation of bile, by furnishing a copious supply of ill-digested chyle, abounding with the elements whence bile is formed;* while, at the same time, the debility which these viscera experience extends itself to the gall-ducts and bladder; and the emulgent operation, usually produced by a healthy and active function of the duodenum, no longer takes place, or, if at all, in a lesser degree. *The accumulation of mucus on the internal surface of the duodenum may also obstruct the mouth of the common duct,* and prevent the flow of bile into the alimentary canal, until this obstruction be either overcome or removed."

But enough of hypothesis. The signs of accumulation in the gall-bladder or biliary ducts, cannot always be depended on, especially when viewed separately; but, taken in connexion, Mr. A. thinks they may be duly estimated by an experienced practitioner.

"The earliest symptoms of which the patient generally complains, when he attends to his sensations and state of health, are, clamminess and foulness of the mouth, fauces, and tongue, with a bitter taste, particularly in the morning; a sense of distension and weight at the epigastric region and at the præcordia, frequently with a sense of coldness and sinking in the same situations; slight anxiety; acid and acrid eructations about three or four hours after a full meal, with painful fulness at the epigastrium, and difficult digestion. The patient often complains of headach, pain in the back or loins, uneasiness under the shoulder-blades, fulness and pain in the region of the liver, particularly when pressure is made at the time of his taking a full inspiration; and of aching in his knees, shoulders, and limbs; his countenance being pale, sallow, or muddy, and the conjunctivæ more or less tinged of a yellowish hue. The state of the pulse is different in different cases. It is often slow and full, and sometimes it is irregular in frequency and strength; occasionally it intermits, and not infrequently becomes quick, but oppressed upon the least motion or exertion. The urine is generally high coloured, and depositing a brownish sediment. The stools are often costive, sometimes light or clay-coloured, and frequently tenacious. When the accumulated bile is discharged into the alimentary canal, much constitutional disturbance then generally arises, according to the qualities which this fluid may have acquired from its retention. The pulse now becomes quick, and often irregular; vomiting and purging, with griping, pain, and anxiety, often supervene, sometimes with spasms. Thirst becomes urgent, and the tongue, which was before foul, is now excited, often dry, and its papillæ large, distinct, and erect."

The ultimate *effects* of these accumulations and subsequent overflowings of vitiated bile, will be various in different individuals. In one it will produce simple bilious diarrhœa—in another, sporadic cholera—in a third, simple dysentery, or inflammation of the bowels, or even the stomach, when the bile regurgitates into that organ. In some instances, though not very frequently, the inflammation will be found confined to the duodenum, as was verified by the following case and dissection.

"A female, leading an irregular life, came into hospital complaining of all the symptoms of bilious accumulations of a morbid character, with much debility, a broken-down constitution, quick, feeble, and fluttering pulse, nausea, and vomiting of dark-green bilious matters, slight purging of dark bilious and fluid motions, coldness of the surface, sunken countenance, and pain and anxiety at the pit of the stomach and right side. Blisters were applied to the epigastrium; laxatives with ammonia were given internally, and enemas of an aperient and cordial kind thrown up. She died soon after admission, and the body was inspected within twelve hours after death. Upon examining the alimentary canal from the œsophagus to the rectum, and exposing its internal surface throughout, the duodenum was found highly inflamed from the pylorus to the jejunum, the upper portion of which latter was also inflamed. A part of the duodenum, a little below the entrance of the ducts, was sphacelated. A few red points were observed in the stomach and other parts of the alimentary canal; but these were not more numerous or extensive than what are often remarked in cases of death from diseases in which the functions of the alimentary canal were unaffected. The portal veins were turgid; the liver somewhat enlarged. There was no other morbid appearance."

The presence of vitiated bile in the duodenum sometimes occasions an alarming state of depression and prostration of the vital energies—especially in nervous and melancholic temperaments. Mr. A. thinks that, in those cases where the natural functions of the bowels have been impeded by accumulations of viscid bile, "the irruption of morbid bile is productive of much less constitutional disturbance, and is even beneficial, inasmuch as it detaches this matter from the mucous surface, and leaves it free and unencumbered in the performance of its functions." Cases in illustration of biliary accumulations are detailed, and the author next proceeds to a new section.

### CONGESTION OF BLOOD IN THE LIVER.

This Mr. A. supposes to be a much more frequent occurrence in this as well as in tropical climates than is imagined. He conceives that it is present in the early stage of the majority of febrile diseases—particularly those which are idiopathic—and that it is not generally overcome until after the stage of excitement has been fully formed. The rationale of this state of hepatic congestion is substantially the same as was given by Dr. Johnson many years ago—namely, the peculiarity of the portal circulation, with reference to the general circulation. Mr. Annesley conceives that this hepatic congestion not only plays an important part in fevers and many other inter-tropical diseases, but leads to hepatic inflammation, the great scourge of Europeans in India.

"We have already alluded to the existence of congestion of the liver, during the progress and decline of other diseases. This is particularly remarkable in the history of the dysenteries of India, and in the remittents, intermittents, and continued fevers of that country, and indeed of other intertropical regions. Even in the dissection of those cases which terminate fatally, whether from fever of whatever type, from dysentery, from cholera, either simple or epidemic, or from disorders of the other abdominal viscera, and even in those more particularly affecting the head or chest, great congestion of the vessels of the liver is not infrequently observed. Nor can the appearance be considered more the consequence of death, or of the changes immediately preceding dissolution, than previously existing disorder; for the attentive observer may often remark the signs usually characterising congestion of the liver, during the life of the patient, or may trace an obvious connexion between this condition of the viscus and the disorder of which the patient died."

The anatomical characters of this congestion of liver are well illustrated by plates, which, from the size and price of the work, are unfortunately beyond the reach of the profession generally. We may shortly state that the viscus is usually much increased in size, particularly the right lobe, and in a direction upwards into that side of the thorax, forming a large segment of a circle. The colour of the organ is generally changed by the congested state of its vessels, and seems to depend on the particular sets of vessels which are the seat of this plethora, and also on the absence or co-existence of accumulations of bile in the ramifications of the hepatic ducts.

"In some cases, the surface of the liver is of a darker brown than natural, almost amounting to black, greenish-black, or bottle green, and this deep colour in some instances passes very abruptly into a reddish or light-brown tinge. Sometimes the surface of the congested liver is variously mottled, or marbled, and occasionally it is streaked and clouded, of a yellowish-brown, greenish-black, or yellowish-green hue. These shades of colour are generally more remarkable upon its upper or convex surface, but they are often observed upon the concave surface, and are quite independent of any effects which may have been produced by the bile contained in the gall-bladder. Sometimes the surface of the liver is very dark; and yet, upon cutting into its substance, the subjacent texture is of its usual colour.

"When cut into, the substance of the liver is, however, generally darker than usual, and gives out a large quantity of dark fluid blood: but in regard to fluidity, there is much difference, according to the period which has elapsed from the time of death to that of inspection. In India, where the *inspectio cadaveris* is usually made a few hours after death, the blood is observed, in cases presenting congestion of the liver, of a fluid or semi-fluid, or thick consistence, and of a very dark colour. The portal vessels and the hepatic veins are the seats of congestion, and it is often difficult to say which of the two sets of vessels presents this appearance to the greater extent, or more frequently; but we believe that the hepatic vein is more generally congested in the greater degree. In many cases, the congestion of the blood-vessels and accumulations of bile in the biliary ducts, although existing to a great extent, are insufficient to account for the very great increase of the size and weight of the liver, shewing that these appearances are often connected with augmented size of the viscus, independently of the extent to which they could have increased its bulk, and of any organic disease. On some occasions, congestion and accumulation of bile have been considerable, without any very marked augmentation of size; but more generally, congestion of the blood-vessels, particularly when associated with accumulations of bile in the biliary ducts and gall-bladder, gives rise to increased size of the liver; and such increase is often in relation to the extent to which congestion of the blood-vesels and biliary ducts obtains."

The appearances of the bile are various. Sometimes it is pale, deepening, in different subjects, from a straw colour to an orange—and from that down to yellowish green—green—dark bottle green, &c. In the lighter shades the bile is generally most fluid, and *vice versâ*. Upon making slices of the congested liver, the divided mouths of the distended ducts appear rounded or oval, according to the direction of the incision—and, in some instances, small granular or miliary calculi are found in the ducts. In cases presenting the greatest degrees of congestion and biliary turgescence, the viscidity of the bile appeared to our author to have given origin to the formation of these small calculi in the substance of the liver. The cystic bile, in these states of congestion, is generally of a green colour of various shades and consistences.

The above morbid appearances are often seen accompanying organic diseases of this viscus.

The *symptoms* which denote, in the living body, these congested states of the biliary vessels, cannot be individually depended on. They must be viewed in connexion.

"When, however, the countenance is pale, anxious, inexpressive, sallow, of a dark or muddy hue; when the tongue is covered with whitish or yellowish-white fur, or otherwise loaded; when the bowels are costive, or when the stools are morbid, dark, and watery, with griping and tenesmus; when the digestion is difficult, attended with nausea, or when the appetite is diminished, and the patient complains of pain and oppression at the scrobiculus cordis, particularly after a meal, with flatulence, borborygmi, and oppressed breathing, and a difficulty of filling the lungs to their utmost; when the skin is cool, clammy, and foul, or of a dark muddy tinge, with irregular chills, sometimes approaching to rigors; when pain, fulness, weight, and oppression, are experienced in the region of the liver, and at the epigastrium, or across the shoulder-blades, or beneath the scapula, and have supervened suddenly; when the uneasiness in those situations is increased upon a full pressure and full inspiration; when the pulse is full, slow, and irregular, or when it is quick, but oppressed; when there is headach, restlessness, disturbed sleep, with unpleasant dreams; and when the urine is turbid, or presenting a muddy sediment,—we may infer that congestion of the vessels of the liver is actually present."

It must be remembered that all, or even the majority of these symptoms are not to be expected in the same individual, although many of them may be recognized in different grades. The state of the pulse is very variable, and not to be depended on. Mr. Annesley thinks that, although pain, oppression, weight about the epigastrium, or under the scapulæ characterise, in general, inflammation of the substance of the liver, yet that these are often marks of congestion also—especially when they supervene suddenly, and are attended with many of the symptoms already described. Inflammation does not arise or reach its acmé in a few hours, but congestion may. Neither can pain, he thinks, be always considered indicative of inflammation, since the membranes of the liver are put on the stretch by congestion. The causes of this congestive condition of the biliary organ are those which have been already portrayed—high atmospheric temperature—too much animal food—too highly seasoned dishes—" indolence and insufficient exercise in the open air"—inordinate use of spirituous liquors.

### TORPOR OF THE LIVER.

We were rather surprised to find this section rise in view, after the brilliant doctrine of increased secretion of bile on carbonic acid principles. The torpid condition of this apparatus, however, could hardly have escaped the notice of Mr. Annesley, though both he and his hypothesis manufacturer appears sadly at a loss to account for the phenomenon. After a great deal of physiological and pathological speculation, the meaning of which is far beyond our comprehension, we come to the pith of the business, in the following short passage.

" Torpor of the liver, then, may arise simply from a diminished or exhausted energy of the secreting functions of the organ; and, from this state, complicated with accumulations of bile in the biliary ducts and gall-bladder, and with congestion in the blood-vessels of the organ; the former state of disorder gradually superinducing, and becoming complicated with, the latter derangements."

Torpor of the liver, Mr. A. observes, is generally complicated with dyspepsia—" and not unfrequently originates in that disorder." As the increase of the secretion was accounted for by the diminished production of carbonic acid gas in the lungs, we wonder that Mr. A. and Dr. Copland did not try to connect the diseased secretion with some modification of the same doctrine. Instead of the carbonic theory, the following explanation is given, which indicates that the doctrine of sympathy was not entirely annihilated in their minds.

" Over-excitement, also, of the perspiratory functions, from long-continued marches, fatiguing exercises, and too warm clothing, is not infrequently productive of considerable exhaustion of the secreting actions of the liver, and often disposes it to torpor, venous congestion, and accumulations of bile in the biliary ducts, *upon the slightest exposure to cold, to moisture, to the impression of malaria*; and when the depressing passions are brought into operation, or when hurtful or indigestible matters are taken into the stomach."

Whether those exposures to cold, moisture, malaria, &c. do not influence the cutaneous secretion, we leave to our readers to determine. The symptoms indicative of this torpid state of the biliary organ are next delineated. It is acknowledged that these symptoms are not always so unequivocal as could be wished.

"If, however, we find the patient to complain of want of appetite, drowsiness, with pain over the eyebrows, lowness of spirits and hypochondriacal feelings, dark and high-coloured urine, a costive state of the bowels, and pale or clayey motions, a dark or sallow countenance, wasting of the flesh, slow and painful digestion, with the symptoms noticed in a previous section as constituting diminished function of the stomach, flatulency, particularly of the bowels, without any evident fulness or enlargement in the region of the liver, but with a bitter or disagreeable taste of the mouth, and a loaded state of the tongue, particularly in the morning,—we may reasonably infer that the functions of the liver are inadequately performed; but it is by no means so easily to be determined whether or no such torpor is the result merely of diminished function, or of change of the structure of the organ, unless we are acquainted with the patient's habits and the nature of his former ailments. When the foregoing symptoms occur in one addicted to the use of spirituous liquors, or in one who has resided long in a warm climate, and suffered former attacks of hepatic disease, then the latter alternative may be more reasonably inferred."

After detailing a sufficient number of cases illustrative of this torpid state of liver, Mr. A. proceeds to the treatment of these functional disorders of the biliary apparatus. This may be summed up in a very concise manner. Where plethora exists, and the patients have been living too free, blood-letting is recommended, as the first step—and local depletion afterwards, if necessary. The antiphlogistic system should be strictly adopted, and the bowels to be kept well cleared by purgatives. Where sickness and bilious vomitings obtain, warm water is ordered, and afterwards a brisk dose of calomel. Even when all the morbid secretions are cleared away, Mr. A. recommends a full dose of mercurial at night, with aperient draughts in the morning, with the view of changing the secretions of the liver, and effecting a healthy flow of bile. If the mouth becomes affected, under this treatment, "a healthy state of function of the liver is the more likely to supervene speedily." It is not the object, however, of Mr. A. to affect the mouth by mercury. In many cases, where the rush of vitiated bile into the duodenum occasions distressing symptoms, cordials will be necessary before the purgative plan is put in force. In respect to that form of disorder, which has been termed torpor of the liver, Mr. A. found a full dose of calomel at bed-time, followed by a bitter aperient medicine in the morning, the most beneficial practice, with blisters over the epigastric or hypochondriac regions. After a few days of this treatment, the pilula hydrargyri combined with the pil. alois. cum myrrha, is prescribed at night, with the bitter aperient in the morning. The practitioner is warned against the exhibition of tonics and stimulants for the apparent debility which accompanies these biliary derangements.

This carries us through full half of this immense volume, and to the end of functional disorders of the biliary apparatus. In another article we hope to afford our readers a general view of the remaining chapters dedicated to inflammation and to various organic diseases of the liver. We shall withhold any general observations on the work till the close of our analysis, hoping by that time to enable every reader to judge for himself. We cannot help, however, again expressing our regret, that the letter-press of this volume should have been so very much expanded by disquisitions that might have been spared, and by didactic precepts far too much spun out. It is probable, indeed, that the talented author may think it much better to be needlessly minute than unsatisfactorily brief. On this point he may be right and we may be wrong.

